# Case Report: Allergic Bronchopulmonary Aspergillosis Revealing Asthma

**DOI:** 10.3389/fimmu.2021.695954

**Published:** 2021-06-22

**Authors:** Houda Snen, Aicha Kallel, Hana Blibech, Sana Jemel, Nozha Ben Salah, Sonia Marouen, Nadia Mehiri, Slah Belhaj, Bechir Louzir, Kalthoum Kallel

**Affiliations:** ^1^ Pulmonary Department, Hospital Mongi Slim, La Marsa, Tunisia; ^2^ Faculty of Medicine, Tunis El Manar University, Tunis, Tunisia; ^3^ Parasitology and Mycology Department, La Rabta Hospital, Tunis, Tunisia

**Keywords:** allergic bronchopulmonary aspergillosis, *Aspergillus fumigatus*, antifungal therapy, drug toxicity, uncontrolled asthma

## Abstract

Allergic bronchopulmonary aspergillosis (ABPA) is an immunological pulmonary disorder caused by hypersensitivity to *Aspergillus* which colonizes the airways of patients with asthma and cystic fibrosis. Its diagnosis could be difficult in some cases due to atypical presentations especially when there is no medical history of asthma. Treatment of ABPA is frequently associated to side effects but cumulated drug toxicity due to different molecules is rarely reported. An accurate choice among the different available molecules and effective on ABPA is crucial. We report a case of ABPA in a woman without a known history of asthma. She presented an acute bronchitis with wheezing dyspnea leading to an acute respiratory failure. She was hospitalized in the intensive care unit. The bronchoscopy revealed a complete obstruction of the left primary bronchus by a sticky greenish material. The culture of this material isolated *Aspergillus fumigatus* and that of bronchial aspiration fluid isolated *Pseudomonas aeruginosa*. The diagnosis of ABPA was based on elevated eosinophil count, the presence of specific IgE and IgG against *Aspergillus fumigatus* and left segmental collapse on chest computed tomography. The patient received an inhaled treatment for her asthma and a high dose of oral corticosteroids for ABPA. Her symptoms improved but during the decrease of corticosteroids, the patient presented a relapse. She received itraconazole in addition to corticosteroids. Four months later, she presented a drug-induced hepatitis due to itraconazole which was immediately stopped. During the monitoring of her asthma which was partially controlled, the patient presented an aseptic osteonecrosis of both femoral heads that required surgery. Nine months after itraconazole discontinuation, she presented a second relapse of her ABPA. She received voriconazole for nine months associated with a low dose of systemic corticosteroid therapy with an improvement of her symptoms. After discontinuation of antifungal treatment, there was no relapse for one year follow-up.

## Introduction

Fungal pulmonary infections are rare in immunocompetent patients. These infections can be caused by several pathogens such as *Aspergillus*, *Pneumocytis jirovecii* and *Cryptococcus*. However, even immunocompetent patients can be affected by these pathogens. In fact, they can affect patients with chronic bronchopulmonary pathologies such as asthma, cystic fibrosis and chronic obstructive pulmonary disease (1). The most frequent pathogen associated with fungal pulmonary infections is *Aspergillus*, which is a saprophytic mold isolated abundantly from soil, construction sites and hospitals (1, 2). There are three clinical presentations of pulmonary aspergillosis: chronic pulmonary aspergillosis (CPA), invasive pulmonary aspergillosis (IPA) and allergic bronchopulmonary aspergillosis (ABPA) (3). Depending on the interaction between the pathogen and its host, pulmonary aspergillosis can lead to one of these clinical presentations (4). The estimated worldwide global rate of ABPA among asthmatic adult patients is 2,5% (5, 6), however only some cases are reported in Tunisia (7). ABPA is most often associated with severe uncontrolled asthma (5, 6) and drug toxicity is frequently reported during therapeutic management. We report a case of ABPA in a patient with a medical history of an allergic rhinitis, with no respiratory symptoms and who developed drug toxicity to corticosteroids then to itraconazole. Thus, we emphasize the challenges in diagnosing and treating ABPA due to its atypical clinical presentations and significant drug toxicity associated with its therapeutic management.

## Case Presentation

**Figure 1:**
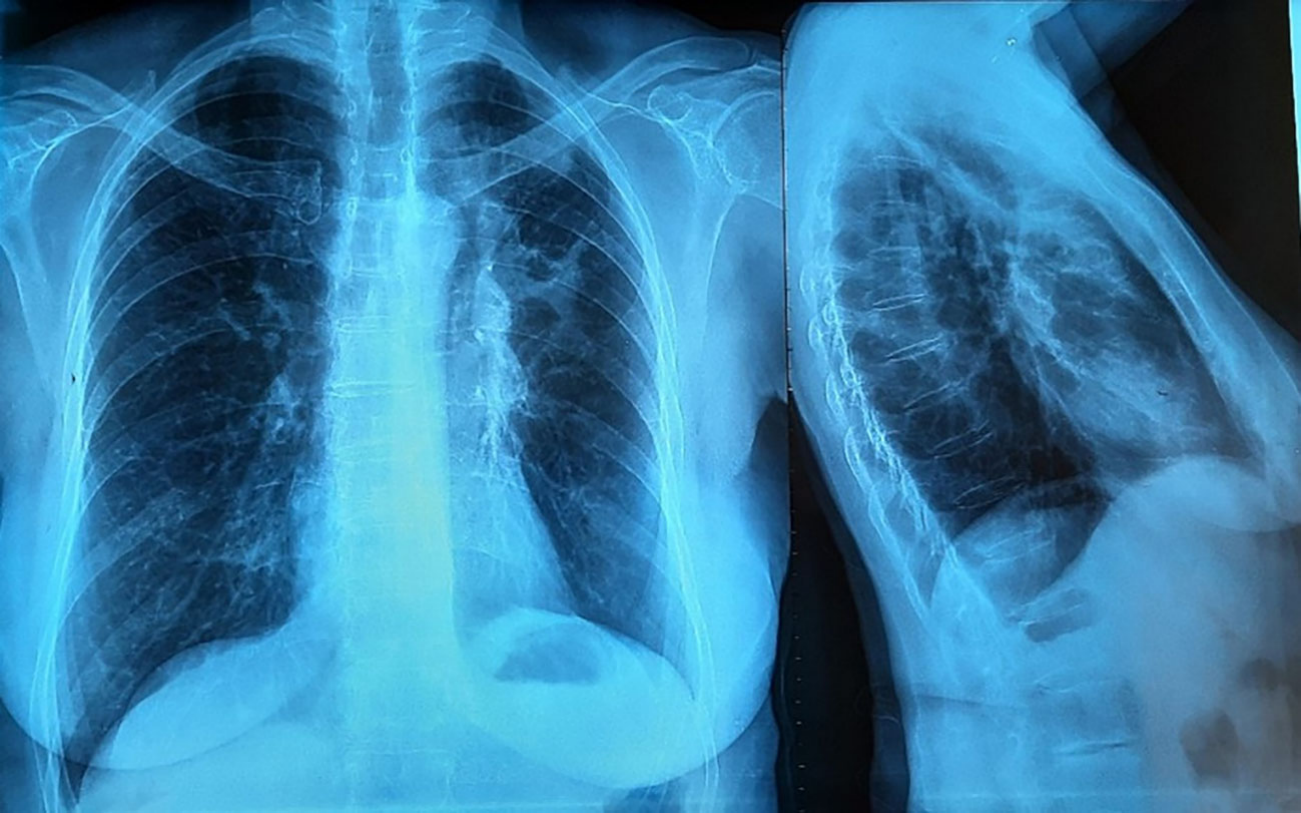
Left hilo-axillary linear opacity associated with retraction signs evoking atelectasis on chest-ray face and profile.

**Figure 2:**
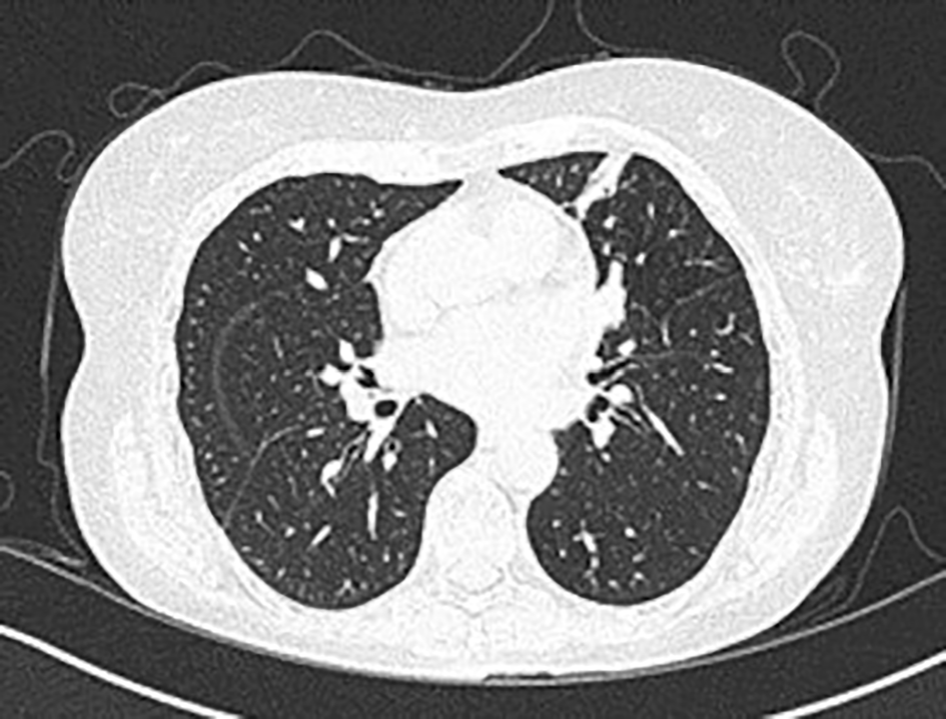
Chest CT scan image showing segmental aerated collapse of the lingula.

**Figure 3:**
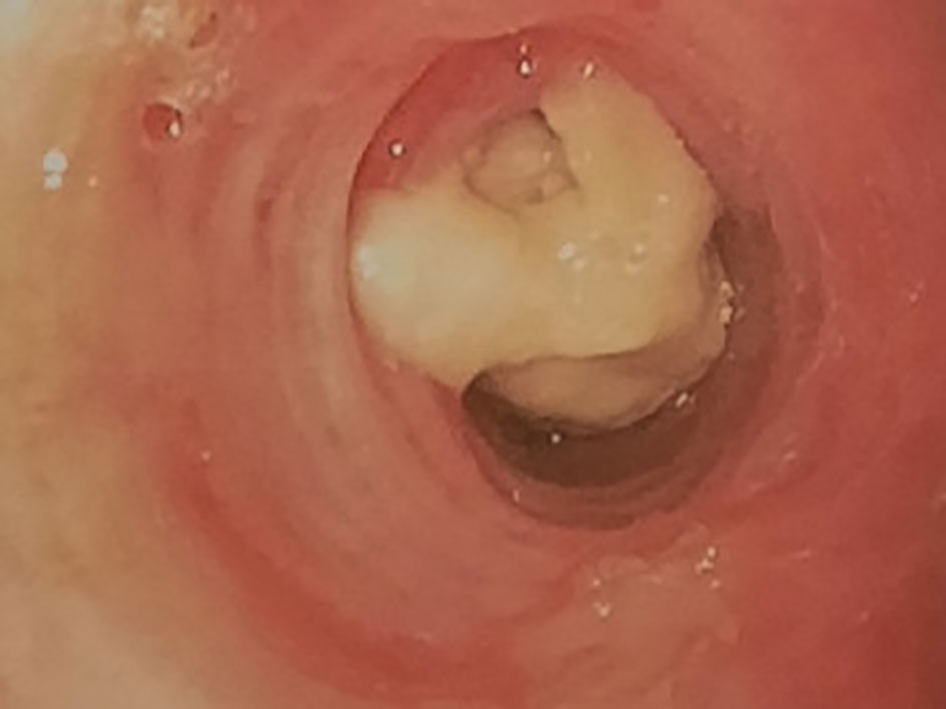
Complete obstruction of the left strain bronchus by sticky greenish material in flexible bronchoscopy.

**Figure 4:**
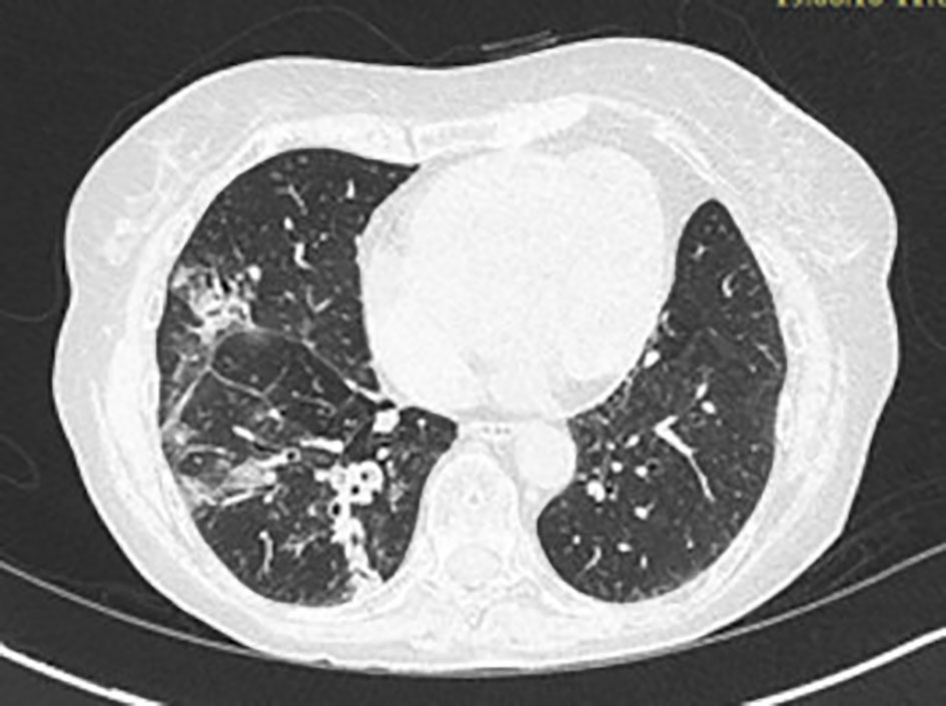
Chest CT scan image showing alveolar opacities associated to bronchiectasis in posterior and medial segment of the right basal pyramid.

A 72-year-old woman, non-smoker, consulted in December 2017, an office-based pulmonologist for acute bronchitis with wheezing dyspnea. In her medical history she reported an allergic rhinitis diagnosed in childhood without respiratory symptoms. She was sensitized to some pollen types which were not specified. She is of French origin and living in Tunisia for thirty years. She had no respiratory nor rhinitis symptoms since she came in Tunisia and until she developed a bronchitis in December 2017. This bronchitis was resistant to symptomatic treatment and short-term systemic corticosteroid therapy. The patient was hospitalized in an intensive care unit for an acute respiratory failure due to her bronchitis. A chest X-ray (face and profile, **Figure 1**) showed a left hilo-axillary linear opacity with retraction signs evoking atelectasis. Her blood tests didn’t show a biological inflammatory syndrome (CRP= 11mg/l; white blood cells= 9780/mm^3^ with an eosinophilic count= 939/mm^3^). Chest computed tomography (CT) confirmed the diagnosis of atelectasis and showed segmental collapse of the lingula and posterior segment of the left basal pyramid with no parenchymatous lesion (**Figure 2**). Flexible bronchoscopy revealed a complete obstruction of the left primary bronchus by a sticky greenish material that could be removed (**Figure 3**). Bacterial culture of this material isolated *Pseudomonas aeruginosa* and the patient received consequently an antibiotherapy associating Levofloxacin and Cefpodoxime for two weeks, with partial improvement in respiratory symptoms. The mycological culture isolated *Aspergillus fumigatus*. Aspergillus serology (IgG) was positive at 12 AU/mL (ELISA,Biorad^®^). Total IgE count was 233 IU/ml (ELFA, Biomérieux^®^). *Aspergillus fumigatus* specific IgE and *Aspergillus* skin testing were not done. During pulmonary function test, the forced vital capacity (FVC) was at 1,97 l (82%) and forced expiratory volume in one second (FEV1) was at 1,60 l (81%). The diagnosis of ABPA associated to an asthma was established. The patient received a high-dose regimen of oral corticosteroids for five weeks (1 mg/kg/day: 80mg of prednisone for one week then 40mg for one week then 20mg for one week then 10mg for one week then 5mg for one week) and an inhaled association of long-acting bronchodilator and a high dose of corticosteroids (salmeterol and fluticasone). When searching for mold exposure in the patient’s medical history, we discovered her gardening activities and that she had multiple indoor plants at her house. In addition to medical treatment, it was recommended that the patient should remove all the indoor plants. The evolution has been marked by the disappearance of respiratory symptoms and of the atelectasis on chest x-rays and the decrease in the eosinophils’ count (282/mm3). However, during the decrease of corticosteroid therapy dosage, she presented a relapse of her respiratory symptoms at the dose of 5mg of prednisone per day. She consulted in March in our department. The itraconazole was prescribed in association with corticosteroid therapy (medium-dose regimen: 0,5 mg/kg/day; 40mg of prednisone for one month then 30mg for one month then 25mg for two weeks then 20mg for two weeks then 15mg for two weeks, then 10mg for two weeks). After four months of anti-fungal treatment, the patient developed a jaundice with an intense deterioration of her general condition. Liver biological tests showed significant hepatic cytolysis (AST= 485 IU/L, ALT= 182 IU/L) and cholestasis (Gamma-GT= 933 IU/L, Alkaline phosphatse= 758 IU/L). After exclusion of the intake of any other treatment associated to drug liver toxicity, the diagnosis of an acute liver failure due to a drug-induced hepatitis associated to the anti-fungal treatment was established. As a result, the itraconazole was immediately stopped. At that time, the titer of *Aspergillus fumigatus* specific IgE was 0.59 KUA/L (FEIA,Thermaoscientific/Phadia^®^), hence the decision to continue inhaled asthma treatment and to continue monitoring the patient. For the next seven months, the patient’s asthma was partially controlled. The patient developed a right hip joint pain and a lameness. The various explorations concluded to an aseptic osteonecrosis of both femoral heads. The patient underwent surgery for her right hip. In May 2019, the patient presented acute bronchitis with mucus produced by coughs. She had persistent respiratory symptoms which were resistant to antibiotherapy and short-term systemic corticosteroid therapy. Chest X-ray showed an atelectasis in the left lower lobe. Flexible bronchoscopy revealed a mucus plug at the left lower lobar bronchus. The middle lobar bronchus had a reduced caliber and was non-catheterizable. Chest CT revealed alveolar opacities associated with bronchiectasis in the posterior and medial segment of the right basal pyramid and lateral segment of the middle lobe (**Figure 4**). During bronchoscopy, the plug has been removed and cultures done. Bacterial culture of this material was negative. The mycological culture isolated *Aspergillus fumigatus*. In vitro susceptibility testing against voriconazole was performed using E-test (Biomérieux^®^). This strain was susceptible to Voriconazole (MIC: 0.38µg/mL). Total IgE count was at 301 IU/ml, eosinophilic count was at 159/mm^3^. The titer of *Aspergillus fumigatus* specific IgE was at 13.90 KUA/L. Voriconazole was prescribed in addition to a step up in the patient’s asthma treatment. The patient received antifungal treatment for nine months in addition to a low dose of systemic corticosteroid therapy (prednisone 10mg/day) and an inhaled long-acting anticholinergic (tiotropium) in association to montelukast and an inhaled association of long-acting bronchodilator and a high dose of corticosteroids. The *Aspergillus fumigatus* specific IgE decreased to 2 KUA/L. The pulmonary function test results improved [FVC=2,38 l (90%) and FEV1 = 1,98 l (96%)]. After discontinuation of antifungal treatment, there was no relapse for one year follow-up.

## Discussion

ABPA is caused by hypersensitivity to *Aspergillus fumigatus* (8). It is frequently associated with severe and uncontrolled asthma or cystic fibrosis. The small conidia of *Aspergillus fumigatus* can easily enter the airways. Exposure to large numbers of conidia may cause ABPA (9, 10), but not all asthmatics develop ABPA despite being exposed to the same environmental factors. This means that other factors play a role in the pathogenesis of ABPA (9). In a genetically predisposed individuals, inhaled conidia of *Aspergillus fumigatus* germinate into hyphae with release antigens that activate the innate and adaptive immune responses (Th2 CD4+ T cell responses) of the lung (9, 10).

When treating ABPA, exposure to molds in the patient’s environment must be investigated to indicate remediation. This is important to prevent relapse after discontinuation of treatment, which was the case for our patient. The first publication about ABPA as an entity was in 1952, from the United Kingdom (11). A decade later, the second case of ABPA was described in the United States (12, 13). The diagnosis of this disease is still difficult, particularly during atypical clinical presentations which was the case of our patient, who had an allergic rhinitis and no prior asthma history or diagnosis. In fact, up to one third of patients with controlled asthma had a relatively asymptomatic ABPA, and the diagnosis is discovered during routine testing (14). Moreover, some clinical presentations are confusing such as the case reported by Savi and al. and where the diagnosis of ABPA was established in a previously healthy male (15). Also, in a nationwide Japan survey, 19% of patients diagnosed with ABPA had no medical history of asthma (16). ABPA is due to an inflammatory pulmonary disorder which often causes non-specific symptoms such as chronic cough, wheezing and recurrent pulmonary infiltration (8). It may be associated to other symptoms such as fever, weight loss, deterioration of general condition, hemoptysis, chest pain and night sweats (17). Expectoration of yellowish-green lumps of mucus is characteristic of ABPA and can be observed in half of the cases (8) which was the case of our patient. The chest X-ray may be normal in the early stages of the disease and can reveal some abnormalities such as “Tramline shadows” and “finger-in-glove opacities” which are temporary patterns corresponding to bronchial wall edema and thickening; “Toothpaste shadows” which are also transient and indicate mucus plugs within bronchi; “parallel line shadows” which appear when the mucus plugs are expectorated (18, 19). In the case of our patient, the chest X-ray showed a linear opacity due to an atelectasis during the first consultation and during the relapse. The chest CT confirmed the diagnosis of segmental collapse during the first consultation and showed bronchiectasis during the relapse. Flexible bronchoscopy has an important place particularly in patients with atelectasis to rule out malignant etiologies. It also allows bacteriological and mycological samples. Sputum or bronchial fluid cultures are positive for *Aspergillus* in nearly 40–60% of cases (20, 21). The presence of *Aspergillus fumigatus* in the sputum culture is not sufficient to confirm the diagnosis of ABPA as this fungus is human saprophyte and can be present in other pulmonary diseases (9, 10). Sputum cultures can contribute to the diagnosis by isolating *Aspergillus* and by performing *in vitro* antifungal-susceptibility testing and molecular testing for resistance, of the isolated strains (9, 10, 22). In a study involving 13 countries from four continents, 6% of the 2026 isolates of *A. fumigatus* were triazole resistant using molecular tools (23). This resistance prevalence varies in its geographic distribution. For example, in a French study, the prevalence of *Aspergillus fumigatus* azole resistance in patients with cystic fibrosis was detected in 6.8% of cases (24). However, in an English study, the azole resistance prevalence was higher. Resistance to at least one azole antifungal drug was confirmed in 13.2% of included patients among whom 16.2% had cystic fibrosis **(**TR_34_/L98H was identified in 27.3% of azole-resistant isolates) (25). Furthermore, the association of *Pseudomonas aeruginosa* and *Aspergillus fumigatus* has been reported in the literature. Both microbes are responsible for considerable morbidity and mortality particularly in patients with cystic fibrosis, among whom the co-infection accelerates the lung disease progression (26). In fact, metabolite exchange and intermicrobial competitions between both germs have been studied to better explain the important morbidity and mortality due to their association (27).

When the diagnosis of ABPA is suspected, some biological investigations are used for the diagnosis and monitoring of ABPA. The relevant tests are eosinophil count, total serum IgE level, serum IgE antibodies specific to *Aspergillus fumigatus* and serum precipitins or specific IgG against *Aspergillus fumigatus* (9, 14). First, blood eosinophil count should be checked and a level over 500 cell/L can help to establish the diagnosis. Our patient had an elevated eosinophilic count (939/mm3). However, high eosinophil counts can be detected in many other diseases and normal levels are reported in patients with ABPA receiving corticosteroids (14). It is known that the pulmonary eosinophilia is far greater than in peripheral blood; thus, a low eosinophil count does not exclude ABPA (9, 28). The measurement of the serum total IgE level is an accurate and important test for the diagnosis and the follow-up of ABPA (10). Active ABPA is generally excluded when serum IgE is normal (9, 10). For the cut-off value of IgE level that should be used in the diagnosis of ABPA, there is no consensus, and it remains uncertain (9). In addition, the reported IgE values in different units (IU/mL, ng/mL) could lead to false interpretation (9). Some laboratories employ 417 IU/mL as a cut-off value, while others use a value of 1000 IU/mL (29). So, a validation of the IgE cutoff value across all populations is required since it could be influenced by both risk of exposure to *Aspergillus* antigens and ethnicity (9, 18). Despite this, the most sensitive investigation in the diagnosis of ABPA is currently the detection of high levels of serum IgE antibodies specific to *Aspergillus fumigatus* (>0.35 kUA/l). This test is also considered the preferred one for screening asthmatic patients for ABPA (6, 9, 10). When our patient had the first relapse, the serum specific IgE were high with a value of 13.9 KUA/L. Although the detection of IgE antibodies specific to *Aspergillus fumigatus* is useful for the diagnosis, it is less helpful in the follow up of patients (10). In addition, serum precipitins or specific IgG against *Aspergillus fumigatus* are detected in 69–90% of cases of ABPA (9, 30). In our case, the patient had positive specific IgG, but the technics used are not equal. In fact, double gel diffusion technique for the detection of *Aspergillus fumigatus*-specific IgG has a limited sensitivity (27%) in the diagnosis of ABPA, whereas, commercial enzyme immunoassays have a sensitivity exceeding 90% (10, 30).

The diagnosis of ABPA is confirmed when the case presentation meets the criteria established in 2013 by the ABPA Working Group of the International Society for Human and Animal Mycology. If total IgE level are over 1000 IU/mL, two among three criteria are sufficient for establishing the diagnosis of ABPA: positive serum precipitins/*Aspergillus fumigatus* IgG, eosinophil count >500 cell/L, chest CT consistent with ABPA (mucus impaction, tree-in-bud pattern, centrilobular nodules, mosaic attenuation (31); high attenuation mucus, pathognomonic for ABPA (9, 32); segmental, lobar and total lung collapse due to mucus plugs (33–35); central or peripheral bronchiectasis). Patients with a total IgE levels under 1000 IU/mL, who, otherwise, meet all the remaining criteria are also diagnosed with ABPA (9). This is the case for our patient who had a total IgE level under 1000 IU/mL. In the different cases reported in Tunisian patients, total IgE level was also under 1000 IU/mL (7). Patients with uncontrolled asthma and positive skin prick test to *Aspergillus* or IgE sensitization to *Aspergillus* and who have a total IgE levels under 1000 IU/mL, without meeting all the other criteria may be diagnosed with severe asthma with fungal sensitization. A normal total IgE level or a negative screening test in a glucocorticoid-naïve patient potentially excludes the diagnosis of ABPA (9).

Different therapeutics have shown their efficiency in the treatment of ABPA. Glucocorticoids are the first molecules to be used. A randomized trial showed that the medium-dose regimen and high-dose regimen are both effective against ABPA with less side effects for the medium-dose regimen (36). In the medium-dose regimen, prednisolone is prescribed in monotherapy for a total duration of three to five months (0.5 mg/kg/day for two weeks, then on alternate days for eight weeks, then 5 mg less every two weeks) (37). When a patient is on glucocorticoids and still has recurrent exacerbations or worsening pulmonary function test or become glucocorticoid-dependent, antifungal therapy could be added (37, 38). In our case, the patient presented a severe complication of glucocorticoid treatment which is aseptic osteonecrosis of both femoral heads requiring surgery. Itraconazole is usually used with or without glucocorticoids for at least six months, at a dose of 200 mg twice a day (38). It requires frequent liver enzymes level monitoring because of its toxicity (38). In fact, itraconazole can cause liver toxicity which was the case of our patient. Other oral azoles such as voriconzaole and posaconazole are also effective in ABPA and can be used when itraconzaole is toxic or contraindicated (39). However, when there is a drug toxicity due to one molecule of azole, there is a risk of a cross-azole toxicity. So alternative approaches to antifungal treatment, in ABPA, that avoid systemic effects were tested and inhaled amphotericin B has been explored with varying results in uncontrolled studies (40, 41). In our case, inhaled amphotericin B was not available and voriconazole was used without a cross-azole toxicity. It led to remission without relapse after discontinuation of antifungal therapy. Furthermore, omalizumab has also proven its efficacy in ABPA, compared to long-term glucocorticoids and it can be administered even in cases with high level of IgE (42). In the case of acute lung collapse, broncho-alveolar lavage during rigid or flexible bronchoscopy helps the lung re-expansion and significant improvement of ABPA symptoms (33, 43). For patients with thick sputum, chest physiotherapy and nebulized hypertonic saline solution improve the symptoms (44, 45). Patients should be examined every two months with chest radiography and total serum IgE levels until remission (9). Exacerbation is confirmed when the baseline total IgE levels doubles with clinical or radiological deterioration (9). Response to therapy is defined by a minimum of 25% decrease in total IgE levels with clinical and radiological improvement and remission is confirmed when the patient has no exacerbations for at least six months after stopping all therapeutics (9). However, it has not been demonstrated that there are benefits of treating ABPA diagnosed on routine investigation in asymptomatic patients with well controlled asthma. Long-term prognosis of patients with ABPA is still not clear (46). But early detection of the disease and prescription of treatments lead to a good prognosis (47). Untreated patients progress to irreversible lung fibrosis and respiratory failure (48).

## Conclusion

We report a case of ABPA occurring in a woman with a prior history of atopic rhinitis but without known history of asthma. She was exposed to a high indoor and outdoor fungal load. We emphasize the importance of an early diagnosis in order to prevent long-term morbidity associated with the irreversible changes that occur with untreated ABPA. This case highlights the challenges of establishing the diagnosis of ABPA and especially the challenges faced during its therapeutic management due to glucocorticoids’ and triazoles’ significant side effects and drug toxicity. Management of ABPA must include mandatory *Aspergillus* exposure remediation to prevent relapse after discontinuation of treatment.

## Data Availability Statement

The original contributions presented in the study are included in the article/supplementary material. Further inquiries can be directed to the corresponding authors.

## Ethics Statement

Written informed consent was obtained from the individual(s) for the publication of any potentially identifiable images or data included in this article.

## Author Contributions

Diagnostic and therapeutic management: HS, HB, NS, NM, and BL. Immunology and mycology investigation: AK, SJ, SM, SB, and KK. Writing, review and editing: all authors. All authors contributed to the article and approved the submitted version.

## Conflict of Interest

The authors declare that the research was conducted in the absence of any commercial or financial relationships that could be construed as a potential conflict of interest.
